# Spanish Bilingual Morphosyntactic Development in Bilingual Children With and Without Developmental Language Disorder: Articles, Clitics, Verbs, and the Subjunctive Mood

**DOI:** 10.1044/2023_JSLHR-23-00091

**Published:** 2023-08-16

**Authors:** Anny Castilla-Earls, Juliana Ronderos, Lisa Fitton

**Affiliations:** a Department of Communication Sciences and Disorders, University of Houston, TX; b Department of Speech, Language and Hearing Sciences, Boston University, MA; c Department of Communication Sciences and Disorders, University of South Carolina, Columbia

## Abstract

**Purpose:**

The purpose of this study was to examine the growth of previously established clinical markers of developmental language disorder (DLD) in Spanish-speaking bilingual children with and without DLD.

**Method:**

Forty-three bilingual children with DLD and 57 typically developing children were tested 3 times over a 2-year period. Their average age at Time 1 was 5;10 (years;months). All children completed an elicitation task examining the production of articles, clitics, verbs, and the subjunctive mood in Spanish at each time point, in addition to other behavioral testing in Spanish and English. We used growth curve analysis to examine change patterns of the morphosyntactic structures over time.

**Results:**

At the onset of the study, children without DLD produced higher accuracy rates than children with DLD across all morphosyntactic structures. In addition, there was a positive effect of time on all structures. Furthermore, the interaction between time and DLD was statistically significant for clitic pronouns.

**Conclusion:**

In agreement with previous literature on language growth in monolingual children with DLD, bilingual children with DLD showed language growth that was parallel to that of bilingual children without DLD but with significantly lower levels of attainment.

**Supplemental Material and Presentation Video:**

https://doi.org/10.23641/asha.23810820

The purpose of this study was to examine the growth of Spanish morphosyntactic structures such as articles, clitics, verbs, and the subjunctive in Spanish–English bilingual children raised in the United States. Spanish–English bilingual Latinx/a/o children in the United States often learn Spanish at home as a heritage language and English from school and the larger community. In this context, English is not only the majority language with the most opportunities for use but also the language of prestige. In contrast, Spanish is a minority or minoritized language, which is considered to be low prestige, and is often associated with lower socioeconomic status (for a historical review, see Fitzgerald, 1993). Although numerically Spanish is the largest minority language spoken in the United States, the level of support it receives from the larger community is relatively low. This sociolinguistic imbalance has key implications for children who are raised bilingually in that fewer opportunities are available to learn Spanish from the community. For example, even in larger metropolitan cities with a considerable number of Spanish speakers (e.g., Houston metropolitan area), there is limited access to Spanish-language books, movies, or community events. Hence, naturalistic interactions in Spanish are often restricted to a few speakers: the immediate family, and, in some cases, extended family or relatives from a Spanish-speaking country. Although Spanish–English bilingual education and Spanish immersion programs are available, these are limited geographically to areas with larger Spanish-speaking populations.

This sociolinguistic context directly influences the acquisition of Spanish in bilingual children in the United States. Although Spanish is often their first language, bilingual children experience a shift in language proficiency in which Spanish becomes their weaker language, and English takes its place as the dominant language (Lutz, 2008). This transition period is associated with the onset of schooling, a period in which exposure to English significantly increases. Spanish morphosyntactic development is at the center of this transition, with large variability observed in bilingual children's morphosyntactic productions (e.g., Montrul & Potowski, 2007; Shin, 2018). It is important to note that this shift in language dominance is not due to limitations in language learning capacity but instead seems to be a result of the limited support for Spanish development. Under these conditions, bilingual children develop morphosyntactic structures at a different rate, and their grammatical productions are more prone to errors in comparison to monolingual children (Shin, 2018).

## Spanish Morphosyntactic Language Growth in Bilingual Children

The Spanish morphosyntactic development of bilingual children (also described in the literature as dual language learners, English learners, or multilingual learners) in the United States has been described in terms of two developmental patterns: bilingual acquisition and language attrition. Bilingual acquisition, also known as incomplete acquisition (Montrul, 2008) or differential acquisition (Kupisch & Rothman, 2016), is a framework that describes bilingual children's language acquisition as different from that of monolingual children in both quantitative (e.g., use of specific forms) and qualitative (e.g., characteristics of the morphosyntactic forms) terms. Within this framework, some aspects of morphosyntax either are acquired over a longer period of time (i.e., protracted development) or do not reach full development in comparison to monolinguals. This bilingual acquisition pattern of development compared to monolinguals seems to be an effect of the input being partitioned between two languages and sociolinguistic factors. Therefore, it is not surprising that bilingual acquisition is unique to the sociolinguistic context of the United States. We believe that there is a place for accurate description of the similarities and differences in the language trajectories between bilingual and monolingual children, provided appropriate research questions (e.g., potential differences in clinical markers of developmental language disorder [DLD] to ensure that the correct norms are used during the diagnosis process). However, comparative frameworks that use a monolingual reference group to define normal language acquisition as standard practice limit our ability to understand bilingualism. Therefore, in this study, we use the term *bilingual acquisition* to describe the unique characteristics of these children who are learning two languages.

An example of the unique characteristics of bilingual acquisition is the work of Montrul and Potowski (2007), who examined the correct production of articles (gender agreement) and adjectives (gender assignment) in a cross-sectional study with bilingual children in Chicago. Their results showed that older bilingual children generally make fewer article errors than younger bilingual children but significantly more errors than younger Spanish monolingual children. A large part of the evidence supporting bilingual acquisition originates from adults who learn Spanish while growing up in a context where Spanish was the minority language. These adults, known as heritage speakers, tend to have inflectional morphology variations (e.g., gender agreement, aspect, and mood) that are unique to their bilingual environment (e.g., Montrul, 2018; Viner, 2016). However, studies examining bilingual acquisition tend to focus on adults and to be cross-sectional in nature, which limits the inferences that can be made about changes within children over time. Therefore, it is unknown if the reported bilingual effects are group differences between individuals or if the development of certain morphosyntactic structures indeed follows a unique trajectory in comparison to monolinguals.

Language attrition, also known as language loss, refers to an observed decline of skills previously acquired, as evidenced in production. This linguistic phenomenon has been documented in bilingual children's morphosyntactic productions. For example, Anderson (1999) documented language attrition for articles in a detailed longitudinal study of two typically developing bilingual children named Victoria and Beatriz. At the onset of the study, Victoria was aged 4;7 (years;months) and Beatriz was aged 6;7. Initially, both children's spontaneous productions showed mastery of articles, with less than 5% of productions in error. However, this accuracy declined, and 2 years later, both children's language contained considerably more article errors, mainly of gender agreement, with error rates closer to 25% for Victoria, the younger child. In addition, both children produced shorter utterances over time, indicating a decrease in their syntactic complexity. The simultaneous increase in article errors and decrease in utterance length suggest that these children were experiencing Spanish language attrition. More recently, Castilla-Earls et al. (2019) examined the longitudinal change in the percentage of grammatical utterances (PGU), a broad measure of grammaticality extracted from spontaneous language samples from 1,080 bilingual children. In this study, bilingual children with typical language skills produced stories in Spanish with a PGU of 94% at the age of 5 years. This PGU in Spanish showed a decline for children who were in English-only instruction (65% at the age of 9 years) and for those in bilingual instruction programs (80% at the age of 9 years), which suggests that there was some attrition in their overall grammatical skills. However, unlike Anderson (1999), this study used a broad measure of grammaticality and, therefore, did not document the specific types of morphosyntactic structures that were affected by language attrition.

It is important to note that the manifestations of both bilingual acquisition and language attrition are not expected to affect all morphosyntactic structures. Instead, it seems that there are morphosyntactic structures (e.g., gender agreement, aspect, and mood) that are more vulnerable to attrition and bilingual effects than other parts of the grammar (e.g., word order; Montrul, 2018). Longitudinal data can provide more compelling evidence of language change and can help tease apart instances of bilingual acquisition or language attrition. Additionally, understanding language change in bilingual children is crucial for the differentiation between typical bilingual development and DLD.

## DLD in Spanish-Speaking Bilingual Children

Bilingual children with DLD exhibit difficulties learning language(s), with particular problems in the area of production morphosyntax (Bishop et al., 2016; Leonard, 2014). These morphosyntactic difficulties are evident in both their spontaneous and elicited language and in all languages spoken by the child (Bedore & Leonard, 2001, 2005; Gutiérrez-Clellen & Simon-Cereijido, 2007). Crucially, the specific morphosyntactic difficulties associated with DLD are language specific. For example, the most salient morphosyntactic difficulties for English-speaking monolingual children with DLD are found in the verb phrase and are related to tense marking (e.g., regular past, third person singular present, copula BE, and auxiliary BE; Rice, 2013; Rice et al., 1998). However, in Spanish-speaking monolingual children with DLD, difficulties are present within both the noun phrase (e.g., articles and clitic pronouns) and the verb phrase (e.g., person agreement, tense, and mood; e.g., Bedore & Leonard, 2001, 2005; Castilla-Earls et al., 2018; Grinstead et al., 2013a; Simon-Cereijido & Gutiérrez-Clellen, 2007). It is generally understood that being bilingual does not exacerbate the grammatical difficulties seen in children with DLD. Instead, bilingual children with DLD exhibit grammatical difficulties specific to each language when using that language in a way that is comparable to monolingual children with DLD (Paradis, 2016).

Articles, clitic pronouns, verbs, and the subjunctive mood are considered clinical markers of DLD in Spanish-speaking children. Specifically, typical development (TD)–DLD group differences have been documented for articles (e.g., Anderson & Souto, 2005; Bedore & Leonard, 2001; Morgan et al., 2013), clitics (e.g., Bedore & Leonard, 2005; Jacobson & Schwartz, 2002; Simon-Cereijido & Gutiérrez-Clellen, 2007), verbs (e.g., Grinstead et al., 2013; Jackson-Maldonado & Maldonado, 2017; Sanz-Torrent et al., 2008), and the subjunctive (e.g., Castilla-Earls et al., 2018, 2021; Morgan et al., 2013). In addition, these structures have been found to have good diagnostic accuracy for identifying monolingual and bilingual children with DLD (Castilla-Earls et al., 2020, 2021). Table 1 summarizes the results of two studies examining the diagnostic accuracy of articles, clitics, verbs, and the subjunctive mood in monolingual and bilingual children with and without DLD using an elicitation task (Castilla-Earls et al., 2020, 2021). We used the data in this table to provide an average baseline of performance on the morphosyntactic structures of interest in this study. Interesting aspects of the data, including small differences in age between these groups, need to be taken into account. First, both monolingual and bilingual children show difficulties on these structures when using this elicitation task. Second, these data suggest that, for all groups, articles are the easiest structure and the subjunctive is the hardest. Third, all four morphosyntactic structures were very difficult for 4-year, 10-month-old bilingual children with DLD, who showed accuracy rates at or below 26% for all structures. The lowest levels of accuracy were observed for clitics (12%) and the subjunctive mood (8%). Lastly, monolingual children with DLD who were, on average, about 1 year older than the bilingual children with DLD had greater accuracy than their bilingual counterparts. This difference in accuracy rates was also observed between the two groups of children with TD who were more comparable in age.

**Table 1.** Summary of results from the studies of Castilla-Earls et al. (2020, 2021).

Grammatical structure	Mon TD Age *M* = 5;8 *n* = 25	Bil TD Age *M* = 5;6 *n* = 33	Mon DLD Age *M* = 5;5 *n* = 25	Bil DLD Age *M* = 4;10 *n* = 33	*t* test
Articles	96% (11)	69% (26)	70% (22)	26% (25)	a, b, c
Clitics	84% (15)	64% (23)	33% (27)	12% (18)	a, b, c
Verbs	78% (15)	65% (21)	50% (22)	26% (17)	a, b, c
Subjunctive	72% (31)	55% (31)	34% (32)	8% (17)	a, b, c

*Note.* Data from the studies of Castilla-Earls et al. (2020, 2021). Letters in the *t* test column indicates a statistically significant difference between the following pairs: a = Mon TD versus Bil TD, b = Mon DLD versus Bil DLD, and c = Bil TD versus Mon DLD. Age *M*: years;months. Mon TD = monolinguals with typical language development; Bil TD = bilinguals with typical language development; Mon DLD = monolinguals with developmental language disorder; Bil DLD = bilinguals with developmental language disorder.

In summary, we currently have evidence that Spanish-speaking children with and without DLD have difficulties with articles, clitics, verbs, and the subjunctive at around the age of 5 years. These structures also seem to be vulnerable to bilingual effects and/or language attrition, as indicated by the differences in performance between monolingual and bilingual children with and without DLD. However, the longitudinal development of these morphosyntactic structures for bilingual children in the United States remains unclear.

## Language Growth in Children With DLD

Most studies examining language growth in children with DLD have been conducted on monolingual English-speaking children (Conti-Ramsden et al., 2012; Goffman & Leonard, 2000; Norbury et al., 2017; Rice, 2013; Rice et al., 2006, 2010). The consensus among the various studies is that monolingual children with DLD significantly differ from children with TD in their overall performance on language tasks, with children with TD performing higher than children with DLD, but not on their rate or trajectory of growth. In other words, although the onset the certain language skills is delayed for children with DLD in comparison to children with TD, both groups show the same pattern and rate of growth. Regarding morphosyntax specifically, English-speaking monolingual children with TD master tense-marking morphemes by the age of 4 years, but this does not occur for children with DLD until after the age of 7 years (Rice et al., 1998). Importantly, both groups of children follow the same growth trajectory, an S-shaped curve, and grow at the same rate. It is estimated that children with DLD are approximately 2 years behind typically developing children for tense marking (Rice, 2012, 2013). Therefore, the production of tense marking is a clinical marker of DLD early in development, but the gap in performance between children with DLD and TD closes over time. For instance, monolingual children with DLD no longer have production difficulties with tense marking after the age of 9 years, but they continue to lag in other language skills (e.g., grammatical judgments; Rice, 2013).

To the best of our knowledge, there are no longitudinal studies investigating the growth patterns of clinical markers of DLD in Spanish–English bilingual children. A crucial point worth noting is that bilingual children learn two languages while growing up, with large variations in the timing and amount of exposure to both languages over time that could affect the growth patterns of the languages. Recall that Spanish–English bilingual children often learn Spanish at home as their first language and English at school and in the community as their second language. Would the same pattern of growth observed in monolingual children with DLD, in which there is a delayed onset of the acquisition but similar growth trajectories, be observed in both languages of the bilingual child with DLD? We believe this might be the case if variations in bilingual experience are considered. For example, growth patterns in Spanish, the home language, should be similar to what is observed in monolingual children if enough language support is provided for the maintenance of Spanish. However, it is also possible that bilingual effects or language loss play a role in the growth of these structures, as previously discussed, resulting in different growth patterns for the home language for all children. For English, we expect to see that the onset of skills appears later for most children since they learn English when schooling starts, with the onset of skills for children with DLD delayed in comparison to children with TD.

## This Study

The purpose of this study was to examine the growth of articles, clitics, verbs, and the subjunctive in Spanish in two groups of children: bilingual children with typically developing skills and bilingual children with DLD. These morphosyntactic structures seem to be vulnerable to bilingual acquisition and/or language attrition and are also clinical markers of DLD in Spanish-speaking children. Longitudinal studies of language development that examine these morphosyntactic structures are highly needed to better explain the morphosyntactic attainment in Spanish for bilingual children with and without DLD.

In this study, we employed the same task used in the study of Castilla-Earls et al. (2020, 2021) to elicit the production of articles, clitics, verbs, and the subjunctive. This task was developed to elicit the production of grammatical structures that are clinical markers of DLD in Spanish. The initial version of the task, which included articles, clitics, adjectives, and plurals, showed developmental changes in the production of these structures between 3- and 4-year-old monolingual children (Castilla, 2008). This task was updated to also elicit the production of verbs and the subjunctive. Using this updated task, significant differences between children with and without DLD were observed in the production of the morphosyntactic structures examined in both Spanish monolingual children in Mexico and Spanish–English bilingual children in the United States (Castilla-Earls et al., 2020, 2021). In addition to the significant group differences, various morphosyntactic structures in this task, both in isolation and in combination, were shown to have acceptable-to-good diagnostic accuracy to identify children with DLD. In this study, we expand the previous use of this task focusing on diagnostic decision making to the examination of changes over time. To do so, we used a longitudinal design to document within-children (time) and between-children (TD vs. DLD) effects. We target children from 4 through 8 years of age because this age range is often associated with changes observed in the home language that are the result of the transition from home to school.

Because the morphosyntactic measurement scales have not previously been used longitudinally, it was essential to evaluate their consistency and general functioning over time (Daub et al., 2021). In the present work, we aimed to provide preliminary evidence of the measures' reliability to inform future use and interpretation of results. We first examined the internal reliability of the measurement scales to ensure that the task elicits consistent responses between similar items and then evaluated reliability over time, taking into account that we expected changes in participants' performance on the task across the duration of the study. We expected the scales to yield relatively consistent differentiation between individual participants, but not full agreement in individual scores, over time. Therefore, we evaluated longitudinal reliability by examining consistency in the rank ordering of participants (Berchtold, 2016; Kottner et al., 2011). We will refer to this type of reliability as test–retest reliability, henceforth. The following research questions (R) and hypotheses (H) guided this investigation:

1. What is the reliability of the morphosyntactic measurement scales? H1: The items were designed to measure the same construct consistently within each subscale, so it is expected that the task will elicit responses from children in a reliable manner.
2. How does response accuracy for articles, clitics, verbs, and subjunctive mood change over time in bilingual children with and without DLD? Two potential growth patterns could be observed. H2a: If language attrition is occurring, we will observe a pattern of decrease, consistent with the observations of Anderson (1999). H2b: Alternatively, a pattern of increase in accuracy over time could be observed, indicating continued development of language skills but without reaching the acquisition attainment seen in monolingual children, consistent with bilingual acquisition, as posited by Montrul (2008).
3. Are there differences in the growth patterns by group (TD vs. DLD) and grammatical structure (articles, clitics, verbs, and the subjunctive)? H3a: Bilingual children with DLD will lag behind children with TD in all grammatical structures of interest in this study, but the growth rate and pattern will be the same, consistent with findings for English-speaking monolingual children (Rice, 2012, 2013). H3b: Differences in accuracy will be observed between articles and clitics and between verbs and the subjunctive, with articles and verbs produced accurately at a higher rate than clitics and the subjunctive, consistent with cross-sectional studies using the same task (Castilla-Earls et al., 2020, 2021).

## Method

Data collection for this study was approved by the institutional review board (IRB) of the University of Houston. This study was planned as a three–time point longitudinal study over the course of 2 years. Data collection for all children at Time 1 was done in person between the fall of 2018 and the spring of 2019. Data collection for Time 2 was ongoing in person after 12 months (± 3 months) starting in the fall of 2019 but was interrupted when the COVID-19 global pandemic affected in-person data collection efforts in March 2020. Thus, we pivoted to remote data collection, and we administered the remaining Time 2 sessions remotely, approximately 15–18 months from Time 1. All sessions for Time 3 were administered remotely between the fall of 2020 and the spring of 2021, approximately 24 months (± 3 months) after Time 1.

### Participants

Children were eligible for the study if they met the following criteria at the onset of the study: (a) They were between 4;0 and 8;11; (b) they spoke and understood both Spanish and English, based on parental report; (c) their parents spoke Spanish at home (at least as often as English); (d) they passed a hearing screening test; and (e) they scored within normal limits on a nonverbal IQ assessment. Hearing screenings were administered using an otoacoustic emissions device at 1000–4000 Hz. If a child failed the hearing screening, they were administered a pure-tone screening test at 25 dB HL at 1000, 2000, and 4000 Hz. Nonverbal IQ was assessed using the Matrices subtest of the Kaufman Brief Intelligence Test–Second Edition (Kaufman & Kaufman, 2004). Standard scores equal to or greater than 70 were considered to be within normal limits. This set of inclusion criteria resulted in 100 bilingual children who met eligibility for this study.

At the onset of the study, the average age was 5;10 (*M* = 70.1 months, *SD* = 12.0). Children were at least a year older when they completed the assessments at Time 2, resulting in an average age of 7;01 (*M* = 85.0 months, *SD* = 13.6). Participants were assessed again at Time 3, at which time the average age was 7;10 (*M* = 94.0 months, *SD* = 12.8). This resulted in a range of ages from 4 to 10 years for the duration of the study, although most children were between 5 and 8 years old during study participation. As with most longitudinal studies, some attrition occurred during the study. Attrition was approximately 10% of participants year over year, which is within the expected range in longitudinal studies, resulting in 100 participants in Time 1, 90 participants in Time 2, and 80 participants in Time 3. The three main reasons for attrition were: unwillingness to continue with the study after contact (*n* = 2), the family moving outside of the United States (*n* = 1), and inability to contact the participant's family (*n* = 16). In addition, one child at Time 3 did not wish to continue with the Spanish language session, so no data was available for the elicitation task in this study.

At the onset of the study, 45% of the participants were female; despite some attrition, this percentage of girls stayed between 44% and 48% during the other time points of the study. We obtained information about demographics and languages using a parental questionnaire administered at Time 1. Fifty-seven percent of the children had mothers who reported having an education of high school or below. All parents responded that Spanish was used at home, and in 45% of the homes, English was also used. For the families that reported speaking both Spanish and English at home, 67% reported that moms spoke mainly or only Spanish with the child, 56% reported that dads spoke mainly or only Spanish with the child, and 53% reported that the extended family spoke mainly or only Spanish with the child. Approximately 90% of the children were exposed to Spanish at school (one was in Spanish immersion, 75 were in Spanish–English bilingual programs, and 14 were in Spanish–English dual-language programs). The remaining 10 children were attending English-only school programs, although five of these children were also attending an all-day Saturday Spanish school program. Detailed descriptive information for participants is shown in Table 2.

**Table 2.** Demographic information for participants.

Information	Time 1 (*N* = 100)	Time 2 (*N* = 90)	Time 3 (*N* = 80)
	*n* (*%*)	*n* (*%*)	*n* (*%*)
Age: *M* (*SD*)	70.07 (11.97)	85.04 (13.64)	93.04 (12.77)
Gender			
Female	45 (45.0)	40 (44.4)	38 (47.5)
Male	55 (55.0)	50 (55.6)	42 (52.5)
Maternal education			
Elementary school	27 (27)		
High school	30 (30)		
Some college	9 (9)		
Associate's degree	7 (7)		
Bachelor's degree	10 (10)		
Graduate degree	14 (14)		
No response	3 (3.0)		
Language(s) at home			
Spanish-only	55 (55.0)	49 (54.4)	45 (56.2)
Both Spanish and English	45 (45.0)	41 (45.6)	35 (43.8)
Testing modality			
In-person	100 (100.0)	38 (42.2)	0 (0.0)
Remote	0 (0.0)	52 (57.8)	80 (100.0)

#### Language Status Classification

Participants were classified as either having DLD or having typical language development using converging evidence according to best practices in diagnosing bilingual children, as described in the work of Castilla-Earls et al. (2020). We used three clinical indicators suggesting the presence of a disorder and classified children as DLD if they met at least two of the three indicators below:

*Indicator 1*. History of receiving speech-language services. Children who were currently receiving or being evaluated for speech-language services in the school or in a clinic at Time 1 qualified under the speech-language pathologist (SLP) indicator. Forty-six children were receiving speech-language services at the onset of the study, and 54 children were not. We did not collect information about the type of speech-language services these children were receiving (e.g., fluency, articulation).

*Indicator 2*. Qualifying score from standardized assessments at Time 1. Children were administered a battery of standardized language assessments in both Spanish and English as part of this study, including the English and Spanish morphosyntax subtests of the Bilingual English–Spanish Assessment (BESA; Peña et al., 2018) and the Bilingual English–Spanish Assessment–Middle Extension field test version (BESA-ME; Peña et al., 2020) and the Sentence Repetition subtest of the Clinical Evaluation of Language Fundamentals (CELF) for both languages (CELF-4 Spanish and CELF-5 English; Semel et al., 2003; Wiig et al., 2013). The BESA and BESA-ME are standardized assessments used to evaluate the language abilities of Spanish–English bilingual children. The BESA is a commercially available language assessment for children ages 4;0–6;11, whereas the BESA-ME is an experimental measure from the authors of the BESA for children ages 7;0–10;11. Both assessments are normed specifically for Spanish–English bilingual children in the United States. The morphosyntax subtests involve a cloze section and a sentence repetition section that target various grammatical morphemes and structures shown to be clinical markers of DLD in English and Spanish. The best language score in the morphosyntax subtest of the BESA has been found to have adequate-to-good diagnostic accuracy for the diagnosis of DLD, with a sensitivity of 90.9% for 4-year-olds, 89.7% for 5-year-olds, and 96.4% for 6-year-olds and a specificity of 83.2% for 4-year-olds, 84.7% for 5-year-olds, and 89.9% for 6-year-olds (Peña et al., 2018). Initial studies of diagnostic accuracy for the BESA-ME morphosyntax subtest also demonstrate adequate sensitivity and specificity in diagnosing DLD in school-age children. Peña et al. (2020) calculated diagnostic accuracy measures for the BESA-ME morphosyntax in the best language for second and fourth graders (second graders: sensitivity = 90.0%, specificity = 89.0; fourth graders: sensitivity = 71%, specificity = 77.0).

We followed the bilingual administration rules of the BESA and BESA-ME morphosyntax subtests and used the “best language” standard score, the associated 95% confidence interval (CI), and the age-based cutoffs specified in the test manuals. At Time 1, 84 children were administered the BESA, and 16 were administered the BESA-ME. Children who were administered the BESA were considered to have a qualifying score from standardized assessments if the full CI for the best language standard score was below the age-based cutoff. Twenty-nine children had a CI below the cutoff, and 40 children had a CI above the cutoff. For the other 15 children who were administered the BESA, the CI included the cutoff, so we examined the scaled scores of the sentence repetition subtest in the CELF in both Spanish and English to have additional evidence from standardized testing for these children. For 12 of these 15 children, the sentence repetition scaled score of the CELF in both languages was under 7 (1 *SD* below the mean scaled score of 10 for this subtest). These 12 children were considered to have qualifying scores from standardized assessments. The remaining three children who were administered the BESA had scaled scores of 7 or more on the CELF in both languages; therefore, they were not considered to have a qualifying score from standardized assessments. The BESA-ME field version does not have CIs for the standard scores. We followed the same steps for classification as for children who completed the BESA but considered borderline scores to be within half a standard deviation (7 standard points) from the 85 cutoff score. This meant that children were considered borderline if they scored in the 78–92 range. Twelve of the 16 children who took the BESA-ME obtained a standard score over 92, and one had a score under 78. The other three children had borderline scores in the BESA-ME; two of them had sentence repetition scaled scores for the CELF in both languages less than 7 and thus were considered to qualify under standardized testing. Using this approach for standardized testing as an indicator, 45 children obtained scores to qualify under standardized testing criteria, and 55 did not.

*Indicator 3*. Qualifying score from language sample analysis. As our last converging evidence indicator, we used measures from spontaneous language samples elicited through story retell and story generation tasks in both languages. We calculated the mean length of utterance in words (MLUw) and the PGU and used the age and MLUw-based cutoff values for PGU provided in the work of Hernandez et al. (2023). Hernandez et al. used language grammaticality (PGU) to determine the child's best language and model-derived cutoffs for PGU, age, and MLUw to account for different levels of language complexity. This method identified children with language disorder with good diagnostic accuracy (sensitivity = 90.3%, specificity = 86.8). For children older than 7 years, we did not have age-based cutoffs for PGU, so we used 80% grammaticality as the cutoff in their best language following findings from Restrepo (1998). Using this approach, 40 children in the study obtained a qualifying score from language sample analysis, and 60 did not qualify with this indicator.

**Figure 1.**
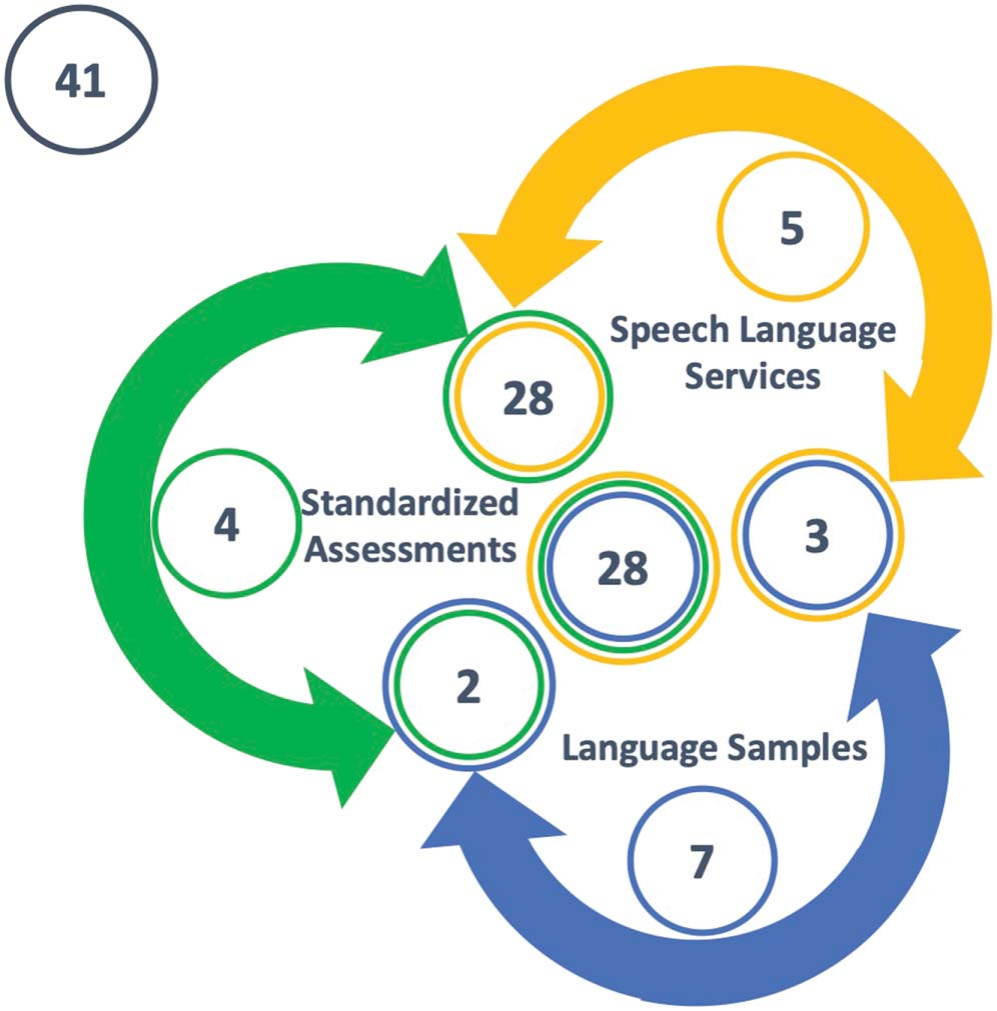
Classification criteria. The diagram represents the classification categorization. Yellow represents children who qualified from the speech-language pathology services indicator. Green represents children who qualified from the standardized assessments indicator. Blue represents children who qualified from the language samples indicator. Black represents children who did not qualify under any indicator. Multiple circles indicate overlapping indicators.

To determine our final language status classification, we combined the evidence from the three indicators. That is, we classified children as having DLD if at least two out of three of our clinical indicators suggested the presence of a disorder. Using this approach, 57 of our 100 participants were classified as TD. Of the 57 children, 41 children did not meet any qualifying criteria, and 16 only qualified under one of the indicators (five children were receiving services, four received qualifying scores from standardized assessments, and seven obtained qualifying scores from language sample analysis). The five children who were receiving services but who were classified as TD tended to have large differentials between their Spanish and English scores in the BESA but had standard scores of 93 or greater in their best language. The remaining 43 participants were classified as DLD. Twenty-eight of these 43 children qualified under all three indicators, and 15 children qualified under at least two of the indicators (10 qualified under SLP services and standardized assessments, two qualified under standardized assessments and language sample analysis, and three qualified under SLP services and language sample analysis). Figure 1 summarizes the process we followed for converging evidence language status classification.

### Stimuli

For this study, we used the same elicitation task developed and used in the works of Castilla-Earls et al. (2020, 2021). The original task, which consisted of 48 items, elicited the production of grammatical morphemes and structures challenging for children with DLD in Spanish, including articles, clitics, verbs, adjectives, plurals, and the subjunctive mood. Children were shown a simple picture with some verbal context and asked a question prompt to elicit the targeted grammatical structures. Since the focus of this study was to analyze performance for articles, clitics, verbs, and the subjunctive mood, this study focused on accuracy results for the 31 items for these structures and excluded items targeting plurals and adjectives from the analyses. For a detailed description of testing items and coding schema, see Castilla-Earls et al. (2020, 2021) and examples of the items in Supplemental Material S1. Two training items for each structure were administered to ensure children understood the task. For articles, the targets were one singular feminine, four singular masculine, two plural feminine, and two plural masculine. Both definite and indefinite articles were accepted in the responses. For clitic pronouns, the targets were two singular feminine, three singular masculine, two plural feminine, and two plural masculine. Three of the clitic items had animate referents, and the other had inanimate referents. For the subjunctive, there were two “quiere que” (*wants to*) and two “cuando” (*when*) clause types. For the verbs, we targeted person and tense agreement (one first person–singular–preterite, one first person–singular–present, one first person–plural–present, one first person–plural–preterite, one second person–singular–present, two third person–plural–present, two third person–plural–preterite, and two third person–singular–preterite). These targets were chosen using previous evidence of difficulties for Spanish-speaking children with DLD (Bedore & Leonard, 2001, 2005). The target of the task was on the general performance of the structure and not on the various forms (e.g., feminine article vs. masculine article). All responses were scored as correct or incorrect. Incorrect responses included omission and substitution errors (e.g., agreement errors) and unintelligible, unrelated, or no responses.

### Procedure

Recruitment for this 2-year, three–time point longitudinal study was primarily targeted at schools with bilingual programs in the Greater Houston. We worked with SLPs in the schools who would identify eligible bilingual children on their caseloads and send the families information about the study. Interested families received study consent forms and parent language questionnaires at Time 1 to participate for the duration of the longitudinal study. Our objective was to have a similar number of children categorized as DLD and TD to have comparable groups when investigating morphosyntactic growth in these children. Once we received consent from families of children receiving SLP services in the schools, we worked with teachers to identify eligible bilingual children with TD from their classrooms (e.g., those not receiving services). Using the school-to-home communication folders, we sent information about the study to invite families. This process allowed us to recruit typically bilingual peers from the same classrooms and schools as the children receiving SLP services. All participating families received a gift card as an incentive for each session the child completed. Although the study was originally conceived as an in-person study, in-person data collection efforts were halted with the onset of the COVID-19 global pandemic, and we turned to remote online data collection. After approval from publishers and the IRB at the University of Houston, we informed parents of the changes to remote online data collection, including the addition of audio and video recording for all sessions. Families interested in continuing the study provided consent to continue under the new procedures. For this study, all data for the 100 participants in Time 1 were collected in person, Time 2 data were collected partly in person and partly remotely, and all Time 3 data were collected remotely.

At Time 1, children were administered three sessions in person for general cognitive skills, Spanish language skills, and English language skills. For Times 2 and 3, only the two language sessions were administered. Sessions were administered by trained research assistants who were native Spanish–English bilinguals. The cognitive skills session consisted of the nonverbal IQ assessment and additional standardized and experimental assessments of various areas of executive function (e.g., attention, working memory). The language sessions included standardized assessments of language abilities (e.g., BESA and BESA-ME Morphosyntax, CELF Sentence Repetition), language sample elicitation using story retell and generation, and the experimental elicitation task used in this study. Sessions lasted approximately 50 min and were scheduled on separate days within 2 weeks of each other. Session order, as well as the order of assessments within a session, was randomized for each participant. All in-person sessions were conducted in a quiet classroom at the school or in a quiet room in the child's home. Sessions conducted remotely were administered via video conference calls using the Zoom software with either the family's laptop or tablet device. For families who did not have a laptop or tablet to use for the remote session, we loaned them a Chromebook device to use for the duration of the sessions.

The elicitation task used in this study was audio-recorded for in-person sessions and video/audio-recorded for remote sessions. Trained research assistants who were blind to the language status classification of participants transcribed all responses. The first author scored all responses as correct or incorrect. Correct responses included the target grammatical structure used following the grammar rules of Spanish. Incorrect responses included omission and substitution errors (e.g., agreement errors) and unscorable responses (e.g., no response, unrelated response, responses in English). To examine the reliability of the data, a trained research assistant scored 20% of all items independently. The interrater reliability at the item level was 93.6%. This high agreement was considered appropriate evidence that the data were reliably coded as correct or incorrect.

### Analytic Plan

To evaluate the reliability of the grammatical structure measurement scales, we conducted analyses based on classical test theory to measure the internal consistency reliability and the test–retest reliability of each scale (i.e., articles, clitics, verbs, and the subjunctive). The scales were designed to be unidimensional, with each item contributing equally to the total score of its corresponding scale. Each item was binarily scored as correct or incorrect. Total scores were computed for each grammatical form based on the average of the child's responses to each item, such that if the child was not administered a single item this was not counted against their overall score. We examined means, standard deviations, and alpha if dropped for each test item at each time point to assess baseline item functioning. Cronbach's alpha was computed as a measure of internal consistency reliability. Test–retest reliability was measured by obtaining Pearson's *r* correlation coefficients between children's scale scores at each time point. We used this approach to evaluate the scales' capacity to differentiate between children when tested twice under the same conditions (i.e., *reliability* definition by Berchtold, 2016; Kottner et al., 2011). Importantly, we did not expect exact agreement in children's scores across the time points because of the inherent focus of our work on growth. Instead, we generally expected consistency in children's rank ordering and relative scores. Pearson's *r* correlation coefficient allows for the evaluation of test–retest reliability under these conditions (Berchtold, 2016; Kottner et al., 2011). Analyses were conducted in R (R Core Team, 2021) using the psych (Revelle, 2021) and dplyr (Wickham et al., 2021) packages.

To examine the growth of articles, clitics, verbs, and subjunctive mood in bilingual children and potential differences by language status group and structure, we used logistic linear mixed modeling. Models were estimated in R (R Core Team, 2021) using the glmer command with a logit link from the lme4 package (Bates et al., 2015). The outcome of interest was children's binary responses to the various grammatical structure items (0 = incorrect, 1 = correct). We included fixed effect predictors for item type, which were dummy coded to allow for comparison between the grammatical forms (i.e., article, clitic, etc.). Models were respecified with each item type serving as the intercept reference, to allow for examination of the model parameters in relation to each grammatical form. To examine change over time, age of the child in years was included in the model using three predictors: (a) age centered within child at the onset of the study (AgeC = AgeChild_T1_), (b) age centered between children at the onset of the study (i.e., AgeG = AgeChild_T1_ – AgeGroupMean_T1_), and (c) an interaction term between A and B (i.e., AgeC × AgeG), to allow for more precise examination of change over time based on specific child age and the timing of the assessment. Accordingly, the intercept represented the log odds of a correct response for a child of average age at the onset of the study. To examine potential differences between children with and without DLD, language status was dummy coded (0 = TD, 1 = DLD) and entered as a fixed effect predictor of item response.

Interaction terms were examined to evaluate differences in growth rates by grammatical form and by language status group. Significant interactions between child age and grammatical form, or child age and language status, would indicate significant differences in the rate of change in children's item responses by these factors. We also tested for a three-way interaction between language status, grammatical form, and child age. The three-way interaction allowed for the evaluation of significant differences in growth rates by language status and grammatical form. This was particularly important to examine because it allowed for investigation into the nature of grammatical differences observed between children with and without DLD by grammatical form and whether children with DLD exhibited specific patterns of change over time that might be substantially different from those of children with TD.

Models included a random intercept for each child, to account for nesting of test items within children (intraclass correlation coefficient [ICC] = 0.36). This allowed individual children's responses to randomly vary at the first time point of data collection. We also tested a random effect for change over time, modeling a random slope based on age at each time point for each child. The random slope accounted for additional variance in predicting children's odds of a correct response and yielded a significant improvement in model fit compared to child random intercepts alone, based on chi-square testing, χ^2^(2) = 230.08, *p* < .001, and the Akaike information criterion (AIC) being lower for the more complex model (9,032.20 compared to 9,258.28). Accordingly, both random intercepts and slopes by child were included as random effects in the statistical models.

To increase the interpretability of results, the findings are reported as probabilities in the text, and tables present findings in log odds. The R packages sjPlot (Lüdecke, 2023) and sjstats (Lüdecke, 2022) were used to format model results into tables. To obtain probabilities, we first converted results from log odds to odds ratios (*OR*s) using the formula *odds* = *exp*(*log odds*). Probabilities were then calculated for specific values of interest within the data sets, using the formula *Pr* = *odds*/(1 + *odds*). We generated plots depicting findings using the ggplot2 package (Wickham, 2016). For considering the variance explained by the fixed and random effects included in the models, we computed Tjur's *R*^2^ (Tjur, 2009). This value is designed to yield estimates comparable to other *R*^2^ values, with possible values between 0 and 1, but specific to logistic regression models.

To evaluate model fit, we first examined the distributions of the residuals in the DHARMa package (Hartig, 2022) for evidence of over- or underdispersion, outliers, zero inflation, and heteroscedasticity. Substantial deviation between the alignment of observed versus expected distribution of residuals, or variability in the scaled residuals versus rank-transformed model-predicted values, would indicate potential model misfit. For models with no clear indication of misfit, we then conducted comparisons of AIC values, with smaller values being preferred, and chi-square tests of nested models. In general, we treated more parsimonious models as preferred unless significant chi-square comparisons indicated a significantly worse model fit for the simpler model.

## Results

### Reliability of the Grammatical Structure Scales

Cronbach's alpha estimates of internal consistency reliability indicated acceptable-to-good reliability for each subscale according to recommended guidelines. Values above .70 may be considered acceptable, above .80 good, and above .90 excellent (Gliem & Gliem, 2003; Nunnally, 1978). For the articles subscale, Cronbach's α values were .79, .86, and .85 at Time Points 1, 2, and 3, respectively. Item total correlations ranged from .48 to .81 (*M* = .68, *SD* = 0.08), which is within the expected range. Item total correlations of .40 or higher suggest appropriate item functioning (Ladhari, 2010; Loiacono et al., 2002). Test–retest reliability was .71 between Time Points 1 and 2 and between Time Points 2 and 3. Values above .70 are generally considered fair, above .80 good, and above .90 excellent (Cicchetti, 1994; Cicchetti & Sparrow, 1990). For the clitic pronouns subscale, Cronbach's α values were .88, .80, and .80 at Time Points 1, 2, and 3, respectively. Item total correlations ranged from .45 to .82 (*M* = .65, *SD* = 0.08), and test–retest reliability was .68 between Time Points 1 and 2 and .70 between Time Points 2 and 3. For the subjunctive mood subscale, Cronbach's α values were .78, .68, and .75 at Time Points 1, 2, and 3, respectively. Item total correlations ranged from .65 to .85 (*M* = .75, *SD* = .05), and test–retest reliability was .64 between Time Points 1 and 2 and .53 between Time Points 2 and 3. For the verbs subscale, Cronbach's α values were .84, .85, and .87 at Time Points 1, 2, and 3, respectively. Item total correlations ranged from .46 to .77 (*M* = .66, *SD* = .08), and test–retest reliability was .75 between Time Points 1 and 2 and .68 between Time Points 2 and 3.

No evidence of substantial items misfit was identified within the item-level statistics (Loiacono et al., 2002). There were no increases in any of the subscales' Cronbach's alpha values if any items were dropped. Further, item-whole correlations indicated a reduction in scales' internal consistency if any items were removed. These indicators suggested that the items yielded sufficient reliability for inclusion within the data set. Average item scores, standard deviations, and missing data rates are provided in Table 3.

**Table 3.** Individual item characteristics by subscale.

Item	Articles			Item	Subjunctive		
	*M*	*SD*	% Missing		*M*	*SD*	% Missing
a1	0.66	0.47	11.7	s1	0.38	0.49	12.7
a2	0.71	0.45	11.7	s2	0.36	0.48	12.7
a3	0.61	0.49	11.7	s3	0.30	0.46	12.7
a4	0.49	0.50	12.7	s4	0.40	0.49	12.7
a5	0.57	0.50	11.7				
a6	0.75	0.43	11.7				
a7	0.70	0.46	12.7				
a8	0.67	0.47	11.7				
**Item**	**Clitics**			**Item**	**Verbs**		
	***M***	***SD***	**% Missing**		***M***	***SD***	**% Missing**
c1	0.47	0.50	12.7	v1	0.52	0.50	11.7
c2	0.47	0.50	11.7	v2	0.63	0.48	12.7
c3	0.35	0.48	12.7	v3	0.55	0.50	11.7
c4	0.25	0.44	12.7	v4	0.51	0.50	11.7
c5	0.45	0.50	12.7	v5	0.68	0.47	12.7
c6	0.54	0.50	12.7	v6	0.72	0.45	12.7
c7	0.26	0.44	12.7	v7	0.72	0.45	12.7
c8	0.40	0.49	12.7	v8	0.64	0.48	11.7
c9	0.49	0.50	12.7	v9	0.83	0.38	11.7
				v10	0.83	0.38	11.7

*Note.* *M* = mean score on item where 0 = incorrect and 1 = correct; *SD* = standard deviation of the mean item score. % Missing data is based on the 100 participants enrolled and the three time points. Of the 100 participants, 10 did not complete Time 2 assessments and 20 did not complete Time 3 assessments. These values are included in missing data calculations.

### Morphosyntactic Performance

We specified three models to select the best-fitting model to explain our data. First, we examined a model including age predictors (AIC = 8,439.86; see Supplemental Material S2). Second, we examined a model with language status as a predictor (AIC = 8,788.27: see Supplemental Material S3). Third, we examined a model with age predictors, language status, and interactions between grammatical form, age, and language status (AIC = 8,411.17; see Table 4 and Supplemental Materials S4–S6). This full three-way interaction model yielded no evidence of misfit based on examination of model residuals. This model also yielded the lowest AIC compared to previous models. Given these observations, we determined that the model including the three-way interactions yielded the best overall fit to the data.

**Table 4.** Model including interactions predicting responses to items: Articles as reference (log-odds).

Predictors	Log-odds			95% CI lower bound		95% CI upper bound	*p* value
(Intercept - Articles)	0.79			0.40		1.18	< .001
LangStatus^a^	**−1.37**			**−1.96**		**−0.79**	**< .001**
Form: Clitic	**−1.07**			**−1.37**		**−0.77**	**< .001**
Form: Verb	0.05			−0.25		0.35	.749
Form: Subj	**−1.75**			**−2.12**		**−1.37**	**< .001**
AgeC^b^	**0.26**			**0.06**		**0.46**	**.010**
AgeG^c^	**0.51**			**0.25**		**0.77**	**< .001**
AgeC × AgeG	**−0.15**			**−0.25**		**−0.05**	**.002**
LangStatus × AgeC	0.20			−0.09		0.49	.175
Clitic × AgeC	**0.25**			**0.02**		**0.48**	**.034**
Verb × AgeC	0.19			−0.05		0.43	.114
Subj × AgeC	0.25			−0.03		0.53	.085
LangStatus × Clitic	**−0.50**			**−0.97**		**−0.02**	**.039**
LangStatus × Verb	0.08			−0.35		0.51	.728
LangStatus × Subj	−0.09			−0.73		0.56	.788
Clitic × LangStatus × AgeC	**0.48**			**0.13**		**0.83**	**.007**
Verb × LangStatus × AgeC	−0.10			−0.43		0.23	.551
Subj × LangStatus × AgeC	0.03			−0.42		0.49	.882
**Random effects**							
	σ^2^	= 3.29	τ_11_	= 0.11	AIC	= 8,411.17	Observations = 8,171
	τ_00_	= 1.42	ρ_01_	= 0.29	ICC	= 0.36	Marginal *R*^2^ / Conditional *R*^2^ = .194 / .484

*Note.* The reported *R^2^* value is Tjur's *R*^2^. Significant coefficients in the model are indicated in bold text. CI = confidence interval; σ^2^ = residual variance, which for logistic regression is fixed at π^2/3^; τ_00_ = between-child variance (i.e., random intercept variance); τ_11_ = between-child random variance in slopes; ρ_01_ = correlation between random slopes and intercepts; ICC = intraclass correlation coefficient; AIC = Akaike information criterion; Subj = subjunctive.

a Language status, coded as 0 = typical development (TD) and 1 = developmental language disorder (DLD).

b Individual child age at each time point of data collection, with 0 = child age at the first time point.

c Difference between individual child age at time 1 and the average age of all participants at time 1.

Detailed model results with articles serving as the reference group are provided in Table 4. Estimates for the same model, but with the other grammatical forms as the reference, are provided in Supplemental Materials S4–S6. Children with typical language skills were most likely to respond correctly to verb items (log odds = 0.84, 95% CI [0.46, 1.21], *OR* = 2.31, Pr = 69.8%) and article items (log odds = 0.79, 95% CI [0.40, 1.18], *OR* = 2.20, Pr = 68.8%) compared to clitics and subjunctive mood items at the onset of the study. There was no significant difference in children's probability of responding correctly to verbs compared to articles at Time 1. Participants were least likely to respond correctly to subjunctive items (log odds = −0.96, 95% CI [−1.39, −0.53], *OR* = 0.38, Pr = 27.7%) at all ages assessed.

Children of average age with DLD were significantly less likely to respond correctly to all four types of items compared to children with TD of the same age at the onset of the study. For instance, children with DLD had a 35.9% probability of responding correctly to an article item in comparison with children with TD who had a probability of 68.8%. For clitics, children with DLD had a 10.4% probability of correct response, whereas children with TD had a 43.0% probability. Regarding verbs, children with DLD had a 38.7% probability of responding correctly to an item in comparison to children with TD who were at 69.8% probability of being correct. Lastly, for subjunctive mood, children with DLD had a probability of 8.1% to respond correctly to an item compared to children with TD who had a 27.6% probability of being accurate. The difference in the likelihood of responding correctly was significant at all time points for all grammatical forms, though the magnitude of the difference varied by grammatical form and time. Overall, the most substantial differences between children with DLD and those with TD were observed for articles and verbs.

**Figure 2.**
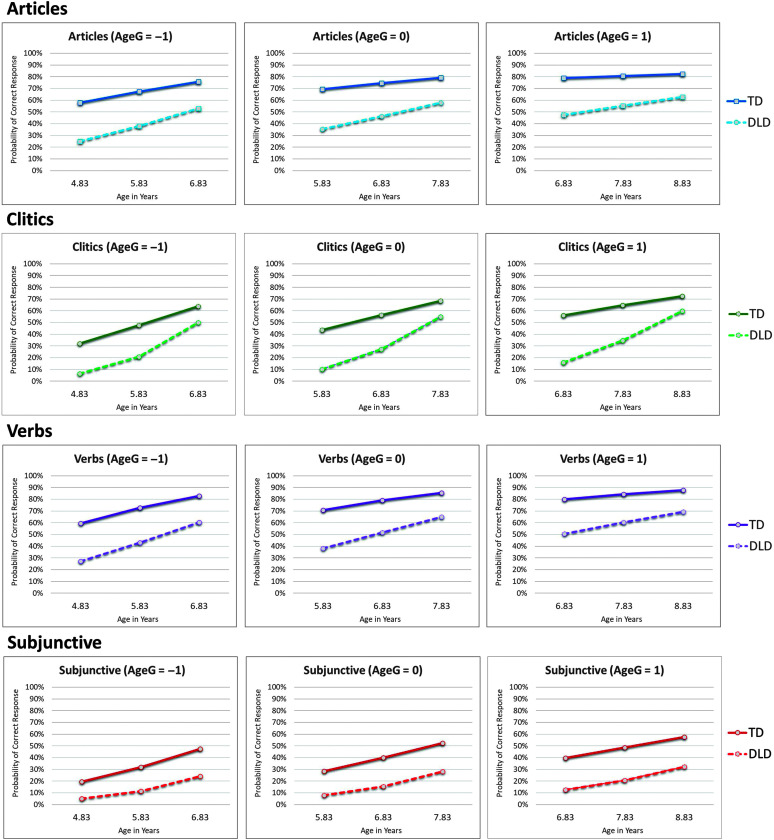
Predicted growth curves for probability of correct responses by item type, language status classification, and age at the onset of the study. AgeG = group-centered age predictor for participants at the onset of the study. AgeG = 0, participants of average age at the onset of the study, 5.83 years old. AgeG = −1, participants one year younger than the average age at the onset of the study, 4.83 years old. AgeG = 1, participants one year older than the average age at the onset of the study, 6.83 years old. DLD = developmental language disorder; TD = typical development.

Overall, both participants with DLD and those with TD were more likely to respond correctly to grammatical structure items as they became older. Further, children who were older at study enrollment, as indicated by the group-centered age predictor (AgeG in all tables), were initially more likely to respond correctly to test items as compared to children who were younger at study enrollment. However, older participants exhibited lesser increases in response accuracy over time, as compared to younger participants. This negative interaction between age at study onset (AgeG) and growth over time (AgeC) was consistent across all four grammatical forms (log odds = −0.15, 95% CI [−0.25, −0.05], Δ *OR* = 0.86). Figure 2 illustrates these findings by grammatical form. The *x*-axes indicate child age, corresponding with child-centered age values. Separate plots are provided to depict differences in the predicted probabilities of correct response based on children's age at enrollment. A value of AgeG = −1 corresponded with 1 year younger than the average age of participants at the onset of the study (i.e., 5.83 years − 1 year = 4.83 years), AgeG = 0 corresponded with the average age at enrollment (i.e., 5.83 years), and AgeG = 1 corresponded with 1 year older than the average age of participants at enrollment (i.e., 5.83 years + 1 year = 6.83 years).

The rate of change observed in children's accuracy of response varied across grammatical forms. For articles, the rate of change was positive and generally stable, aside from among participants who were older at the start of the study (see Table 4). For children with TD of average age at the start of the study, a 1-year increase in age corresponded with an increase in probability of a correct response from 68.8% to 74.1% between the ages of 5.83 and 6.83 years and then to 78.8% at 7.83 years. Differences in the growth of article accuracy between children with DLD compared to children with TD were not statistically significant (log odds = 0.20, 95% CI [−0.09, 0.49], *p* = .175).

Similar trends were observed in participants' performance on verb items (see Supplemental Material S5). For children of average age at the start of the study, a 1-year increase in age corresponded with an increase in the probability of a correct response from 69.8% to 78.4% between the ages of 5.83 and 6.83 years and then to 85.1% at 7.83 years. There was no evidence of differences in growth rates between children with DLD compared to children with TD (log odds = 0.10, 95% CI [−0.17, 0.37], *p* = .477) beyond the difference observed at intercept. Notably, the differences in predicted values for overall accuracy, rate of change, and language status group (DLD vs. TD) between verbs and articles were not statistically significant. Children's likelihood of responding correctly to verb and article items was not significantly different across the study.

Significant differences were evident in children's performance on the clitic pronoun items compared to the other grammatical forms (see Supplemental Material S4). There was a significant positive interaction between participants' language status and growth in accuracy for clitics (log odds = 0.68, 95% CI [0.39, 0.97], *p* < .001), indicating that children with DLD exhibited greater increases in accuracy over time compared to children with TD. However, the gap in performance between children with DLD compared to children with TD did not close by the age of 9 years. Participants with DLD had a 60.8% probability of responding correctly to clitics at age 8;10 compared to the 72.1% probability observed among participants with TD at the same age. This observation reflects the substantial differences in performance observed between children with TD and those with DLD at intercept.

Findings related to children's responses to the subjunctive mood items were generally stable across the duration of the study. As previously noted, participants were significantly less likely to respond correctly to subjunctive items compared to any of the other grammatical forms (see the significant estimates for differences in all three grammatical forms compared to subjunctive in Supplemental Material S6). As observed among the other grammatical forms, children's likelihood of responding correctly did significantly increase as children became older (log odds = 0.51, 95% CI [0.27, 0.75], *p* < .001), with an increase in the probability of a correct response of 27.7% to 38.9% between 5.83 and 6.83 years of age and then to 51.5% at 7.83 years. No significant interactions between the other grammatical forms and age were observed, indicating that children had consistently lower likelihood of responding correctly to subjunctive items at all ages included. There also was not a significant interaction between language status and age (log odds = 0.23, 95% CI [−0.18, 0.65], *p* = .267), indicating that there was no evidence of differences in growth in subjunctive item response accuracy between children with DLD compared to children with TD.

The Tjur's marginal *R*^2^ (Tjur, 2009) value obtained from the full analytic model indicated that language status, grammatical form, age terms, and interactions between these variables accounted for 19.4% of the variance in children's item responses to the Desarrollo Morfologico del Espaþol. The combination of the fixed effects predictors and the random effects, which included random intercepts and slopes for each child, accounted for 48.4% of the overall variance in responses. The random effects estimates indicated substantial between-child variation at intercept (ICC = 0.36; τ_00_ = 1.42) and between-child variation in growth in grammatical production performance over time (τ_11_ = 0.11), beyond that accounted for by the fixed effects.

**Figure 3.**
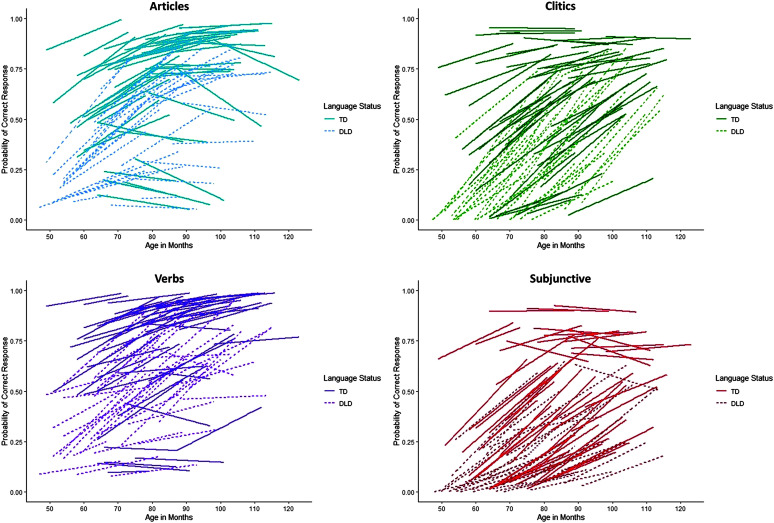
Individual predicted growth curves over time by item type and language status. DLD = developmental language disorder; TD = typical development.

Figure 3 provides a visual depiction of the model-predicted probabilities of correct responses by child for each grammatical form. Predicted responses for children with DLD are indicated by dashed lines, and those for children with TD are indicated by solid lines. This figure shows that there was individual variability in performance between children, which was particularly evident between children in the TD group. Across all grammatical structures, two general patterns of growth were observed. On the one hand, there are children with higher accuracy rates who tended to perform consistently over time. These children were always children with TD. On the other hand, there were children who exhibited increasing performance on the grammatical tasks. Children with DLD, for instance, almost always exhibited increasing patterns of performance across all grammatical structures, with some exceptions. For clitics and the subjunctive, most children with DLD had steep increases in performance over time. Articles was the only grammatical structure that showed that some children decreased in performance over time. These children tended to be those with TD who were older at the start of the study.

## Discussion

The purpose of this study was to examine the growth of four Spanish grammatical forms that have been identified as clinical markers of DLD in a group of Spanish–English bilingual children. First, our results offer evidence of the reliability of the measurement scale we used to examine the production of articles, clitics, verbs, and the subjunctive mood. Second, our results suggest that children with DLD performed significantly lower than children without DLD on all grammatical structures of interest (articles, clitics, verbs, and subjunctive) at the onset of the study. Third, we observed a significant positive effect of time on all structures, with a significant interaction effect for clitic pronouns, suggesting a steeper growth pattern for clitics among children with DLD compared to children with TD.

Findings from the analysis of the grammatical structure scales suggest that the measure was sufficient to measure Spanish articles, clitics, verbs, and subjunctive mood reliably within the present sample. The items were correlated appropriately within each subscale, and there was no evidence that the subscales' reliability would improve if any items were removed. Importantly, the Cronbach's alpha values were generally high for all subscales at each time point, with values in an acceptable range. Only one subscale, the subjunctive mood subscale, yielded a reliability estimate below .70, and this value was .68 at the second time point. Given the variability at this time point due to the onset of the COVID-19 pandemic, and the relative difficulty of subjunctive mood compared to the other grammatical structures, a slightly lower internal consistency estimate for this subscale at this point is not majorly concerning. Therefore, we concluded that the measurement scale used in this study was appropriate to measure the production of the morphological structures of interest.

Results from our comparisons of grammatical performance between children with DLD and those with TD are in agreement with previous studies on monolingual and bilingual acquisition. Prior work has documented statistically significant group differences between children with and without DLD for Spanish articles (e.g., Anderson & Souto, 2005; Bedore & Leonard, 2001; Morgan et al., 2013), clitics (e.g., Bedore & Leonard, 2005; Jacobson & Schwartz, 2002; Simon-Cereijido & Gutiérrez-Clellen, 2007), verbs (e.g., Grinstead et al., 2013; Jackson-Maldonado & Maldonado, 2017; Sanz-Torrent et al., 2008), and the subjunctive (e.g., Castilla-Earls et al., 2018, 2021; Morgan et al., 2013). Because we employed the same task as Castilla-Earls et al. (2020, 2021), we can make specific comparisons to the finding of those studies (refer to Table 1) using the results of our models adjusting for age. Children with DLD at age 4;10 in this study were very comparable to children with DLD in the study of Castilla-Earls et al. (2021) on their average accuracy rates for articles (26% in this study, 26% in Castilla-Earls et al., 2021) and verbs (26% in this study, 26% in Castilla-Earls et al., 2021) but slightly less accurate for clitics (4% in this study, 12% in Castilla-Earls et al., 2021) and subjunctive (5% in this study, 8% in Castilla-Earls et al., 2021). Regarding bilingual children with TD at age 5;6, we also found that children in this study had comparable results for articles (67% in this study, 69% in Castilla-Earls et al., 2021) and verbs (67% in this study, 65% in Castilla-Earls et al., 2021) but less overall accuracy for clitics (39% in this study, 64% in Castilla-Earls et al., 2021) and subjunctive (25% in this study, 55% in Castilla-Earls et al., 2021). Keeping in mind the variability that exists in bilingual children's morphosyntactic performance in general, we were surprised to find similar results, particularly for bilingual children with DLD. The small differences (~25%) in the accuracy production of the subjunctive and clitics for children with TD form both studies might be explained by differences in Spanish proficiency levels and/or language experiences. We should note that these morphosyntactic structures are stable across dialects of Spanish, with the minor exception of the pattern of clitic omission observed in Andean Spanish (Stewart, 1999), but this was not a dialect group included in our study. Overall, these results provide additional evidence that Spanish–English bilingual children with DLD, who receive Spanish instruction at school and speak Spanish at home, produce low accuracy rates for these structures.

As we hypothesized, these bilingual children were, on average, more accurate in their productions of verbs and articles compared to clitics and/or the subjunctive. These results are in agreement with those of Castilla-Earls et al. (2021) and current accounts of morphosyntactic development in Spanish. Within the noun phrase, articles emerge and are mastered before clitic pronouns (Baron et al., 2018; Perez-Leroux & Castilla-Earls, 2016). Within the verb phrase, verb tense/person markers developed earlier than the subjunctive mood, which is considered to be a late-acquired morphosyntactic structure in Spanish-speaking children (Baron et al., 2018; Blake, 1983). Our results are in agreement with the classification proposed by Baron et al. (2018), in which articles and verbs were considered to be easier morphosyntactic structures than clitics and the subjunctive. This is important for assessment practices that attempt to differentiate children with and without DLD in older children. Combining performance on these structures with other language measures, such as grammatical judgments, may continue to accurately classify older bilingual children.

We also hypothesized that children would show an increase in the probability of producing a correct morphosyntactic structure over time for all structures. Our data support this hypothesis for all structures, although differences were observed between structures and between children. An interesting between-children finding related to age was that older children at the onset of the study showed flatter slopes (i.e., slower learning) in comparison to younger children (refer back to Figure 2). We must note here that our longitudinal data set was limited to three time points, and therefore, a quadratic term could not be estimated. A quadratic term could have tested the possibility that the growth in these structures initially increases but slows down at the end. However, the differences in slopes between younger and older children seem to suggest that the growth in these structures initially increased but slowed down at the outset, in agreement with the potential of a quadratic term. These results suggest that the growth of these morphosyntactic structures tends to slow down with age.

The evidence of growth for all grammatical structures on average with older children growing at a slower rate than younger children is, in principle, consistent with bilingual acquisition accounts. At the age of 8 years, for example, bilingual children with TD in our study were predicted to be 79% accurate for articles, 69% for clitics, 86% for verbs, and 54% for the subjunctive. These accuracy rates suggest that the performance on this task at the age of 8 years was comparable to the performance of monolingual children with TD at the age of 5 years (see Table 1) and that these structures in bilingual children are still in a process of acquisition. This difference with monolingual children should not be interpreted as a deficit. In our own view, this bilingual acquisition is a positive characteristic for these children who continue learning morphosyntax in situations where the support of Spanish is limited compared to the support to English. We could either point out that the grammar of these children will not be comparable to the performance of monolingual children of the same age, or we could highlight the learning that occurs in two languages. Curiously, conclusions about monolingual development based on bilingual development are not active research agendas. Although it is well established among researchers that one bilingual is not equal to two monolinguals (see, e.g., Grosjean, 1998), using monolinguals as the reference point and everything that is different in bilinguals described as a gap seems to still permeate scientific discussions in bilingual language acquisition.

This study cannot ascertain whether bilingual children will continue to grow in their accurate production of these structures; therefore, further examination of older children is needed to estimate the development at older ages, particularly because language attitudes and peer influences may affect Spanish skills. The evidence seems to suggest that older children are slowing down in their rate of growth, but that does not signify that continued growth will not take place. Although there is evidence of morphosyntactic variation in adult heritage language speakers, it is difficult to know whether they acquired Spanish but lost some morphosyntax or variation was always present. Across development, individuals' attitudes toward their heritage language may change as a result of environmental influences including peers' perception of the minority language, which will ultimately impact the complexity and the contexts in which they develop their heritage language.

It is important to point out that there was substantial variability between children that needs to be discussed further, as evidenced by the *R*^2^ values obtained. The full analytic model, which included random intercepts and slopes for each child, accounted for 48.4% of the variance in children's responses, whereas the key predictors alone only accounted for 19.4% of the variance in responses. These findings indicate that children vary greatly in their individual responses and rates of growth. Although children showed increases in all morphosyntactic structures as a group, some individual children showed a decline in their overall accuracy, particularly for articles. It is possible that these children were experiencing language attrition, in agreement with the results of Anderson (1999). It is probable that these children are making gender agreement errors that they were not making initially, as seen in Anderson's study; a fuller comparison is not feasible here since error type was not examined in this study. Also, it is interesting to note that these children did not have very high accuracy rates with articles at the onset of the study, which might have made them more prone to language attrition than a child with higher accuracy rates at the onset. Another pattern that emerged from the individual variability is that there were also some children who performed well above average and who maintained that performance over time. These children showed language maintenance over 2 years, suggesting that this is also a potential pattern we should document for bilingual children. In this study, this pattern of high initial performance and language maintenance was only observed for children with TD, but this was probably because children with DLD were in the process of learning the morphosyntactic structures. This pattern of maintenance could depend on certain achievement level to solidify this knowledge over time and the level of contextual support for Spanish. It is important to further investigate what factors might predict language maintenance and language attrition in bilingual children, such as language practices at home, parent's language proficiency, and overall bilingual experience (see Paradis, 2023, for a review).

An important finding in this study is that the gap between children with and without DLD remains the same over time for all grammatical structures tested. This is in agreement with findings for monolingual children in that children with DLD perform significantly worse than children with TD in language tasks but have similar growth rates and trajectories (Rice, 2012, 2013). One difference is that in studies with monolingual children, children with DLD tend to perform closer to children with TD at the age of 8 years on tense marking morphemes (Rice et al., 1998), but in our studies, significant differences were still observed at the age of 8 years for Spanish clinical markers. This might be explained by the bilingual acquisition observed in these children, which seems to have an extended timeline for the acquisition of these Spanish morphosyntactic structures, or the fact that some of these morphological structures, such as the subjunctive, are acquired later in both monolingual and bilingual children (Blake, 1980). The general finding is that the same pattern of language growth observed for monolingual children is observed on average in the home language for the bilingual children in this study when we take into account bilingual development.

### Contextual Considerations

Part of the data for this study was collected while COVID-19 restrictions did not allow in-person testing. We were able to quickly shift to the use of telepractice to continue data collection during Time 2. We purposefully decided to conduct all testing at Time 3 using telepractice so that all children in the study would have in-person data at Time 1 and telepractice data at Time 3. The evidence seems to suggest that telepractice is appropriate for the collection of production data and that it yields similar results to in-person testing (e.g., Ciccia et al., 2011; Manning et al., 2020; Waite et al., 2010). This positive finding is of particular importance for conducting research on marginalized communities, as it may reduce barriers in participating in the research process. In this study, Latino families were able to still participate in our research study, which opens the possibility of increased access to research using telepractice for this underrepresented population.

The findings of this study are important to examine considering the bilingual context in Houston, a major metropolitan area in the United States. Although, in general, the support for English is considerably higher than the support for Spanish, Spanish is a language with strong presence in the community, with approximately 40% of the population speaking Spanish. However, despite this strong presence, Spanish is not considered the language of prestige. It is possible that, at this age, the availability of speakers to provide input in the language is more important the perceptions of its prestige for Spanish learning. Another factor that might have supported the morphosyntactic learning in this study is the availability of bilingual education programs. Most of the children in this study were attending some form of Spanish instruction that might have helped their continued development of Spanish. Spanish instruction seems to offer a protective, although not sufficient, effect on the overall grammaticality of bilingual children over time (Castilla-Earls et al., 2019). The combination of the high the presence of Spanish in the community and the availability of bilingual education programs seems to have provided support for the learning of articles, clitics, verbs, and the subjunctive for this group of children.

### Limitations

There are several limitations that should be acknowledged. First, the children with TD tended to be older than the children with DLD, which may have impacted the precision of the estimates for older children with DLD and younger children with TD. The precision of the estimates might also be impacted by the wide age range in this study and the sample size. Second, we examined the correct production of articles, clitics, verbs, and the subjunctive, but we did not include other developmental information, such as the types of errors, which may offer additional information about the differences in the productions of these morphosyntactic structures between the two groups of children. Third, although the focus of this study was on Spanish development, a limitation of this study is that the growth of English morphosyntax is not described, which could offer important information about language growth in general. Forth, in this study we examined longitudinal reliability. Future studies should examine test–retest reliability. Last, it is important to consider the generalizability of our results. While similar growth patterns can be expected in other bilingual communities across the country, these results may not hold in contexts where sociolinguistic variables and educational programming differ from the children studied here.

## Conclusions

This study provides initial evidence of typical and atypical developmental trajectory of articles, clitics, verbs, and the subjunctive for Spanish-speaking bilingual children. Overall, children with TD performed significantly better than children with DLD at the onset of the study, and this gap in performance did not close over time for the age range examined. In situations of Spanish bilingual development with English language contact, all four morphosyntactic constructions show increases in accuracy beyond the age of 8 years. For both groups of children, articles and verbs seem to be easier morphosyntactic structures than clitics or the subjunctive.

## Data Availability Statement

The data sets generated during and/or analyzed during this study are available from the corresponding author on reasonable request.
